# Expression patterns of fibroblast activation protein and extra-domain B fibronectin in canine malignant tumors

**DOI:** 10.3389/fvets.2025.1719994

**Published:** 2026-02-02

**Authors:** Silvia Dell’Aere, Elisa Maria Gariboldi, Alessandra Ubiali, Roberta Ferrari, Luigi Auletta, Matilde Bocci, Andrea Galbiati, Ettore Gilardoni, Giulia Rotta, Valentina Balbi, Gaia Beatrice Maria Bianchi, Alessandra Verdi, Dario Neri, Samuele Cazzamalli, Damiano Stefanello, Paola Roccabianca

**Affiliations:** 1 Department of Veterinary Medicine and Animal Sciences—DIVAS, University of Milano, Lodi, Italy; 2 R&D Department, Philochem AG, Otelfingen, Switzerland; 3 Philogen S.p.A., Siena, Italy; 4 Department of Chemistry and Applied Biosciences, Swiss Federal Institute of Technology, Zurich, Switzerland

**Keywords:** canine tumor, EDB, extra domain B, FAP, fibroblast activation protein, fibronectin, melanoma, sarcoma

## Abstract

**Introduction:**

Fibroblast activation protein (FAP) is involved in the extracellular matrix (ECM) remodeling and wound healing. Absent in most adult tissues, it is overexpressed by neoplastic cells and/or cancer-associated fibroblasts (CAFs) in several human malignancies. The extra Domain-B of fibronectin (EDB+FN) is a splice variant of fibronectin involved in angiogenesis and tissue remodeling, overexpressed by CAFs and cancer-associated vessels (CAVs) in many aggressive human tumors. This study aims to investigate FAP and EDB+FN expressions in canine tumors and assess their potential as druggable targets in animal patients.

**Methods:**

FAP and EDB+FN expression was assessed by immunohistochemistry on 88 canine tumors, including Soft Tissue Sarcomas [STS], Osteosarcomas [OSA], Hemangiosarcomas [HSA], Apocrine Gland Anal Sac Adenocarcinomas [AGASAC], Mast Cell Tumors [MCT], Lymphomas, and Melanomas, using polyclonal and monoclonal anti-FAP and the L19 anti-EDB antibodies. Expression distribution and intensity were semi-quantitatively scored in neoplastic cells, CAFs, CAVs, and stroma.

**Results:**

FAP was variably expressed in neoplastic cells (79/88), CAFs (79/88), and CAVs (82/88) across all tumor types, but mostly in AGASACs, STSs, and MCTs. The monoclonal antibody presented greater specificity. EDB+FN expression was less present across tumor types, mostly with a vascular staining pattern. Labelling was most intense and consistent in the neoplastic cells, CAFs, and CAVs of melanomas, and to a lesser extent in AGASAC and STS.

**Discussion:**

STS, AGASAC, and MCT could be candidates for FAP-targeted strategies; melanomas are the most promising for EDB+FN-directed therapies. These results support FAP and EDB+FN as targets worth investigating for clinical applications in animal patients.

## Introduction

Fibroblast activation protein (FAP) is a serine protease, expressed as an integral membrane protein or as an intra- and extracellular soluble form (1). FAP is involved in the extracellular matrix (ECM) degradation, thus participating in physiological tissue remodeling and wound healing (1–3).

In humans, FAP is largely missing or minimally expressed in healthy tissues (2), but is overexpressed in several neoplasms such as hepatocellular, pancreatic, esophageal, and ovarian carcinomas, melanomas, and sarcomas, where it can be expressed by neoplastic cells (4, 5) and/or by cancer-associated fibroblasts (CAFs) (5). FAP overexpression by tumor cells has been linked to poor prognosis and the development of metastatic disease (6, 7).

Given its almost exclusive expression in tumoral tissues, FAP has been targeted for cancer diagnosis and therapy (8–13).

In dogs, FAP is physiologically present in endothelial cells (14, 15) and nerve fibers (14) and is expressed by fibroblasts in inflammation (14) and in Canine Idiopathic Pulmonary Fibrosis (16).

FAP expression has been scarcely investigated in canine tumors. FAP expression has been reported in neoplastic cells of canine pulmonary adenocarcinoma (16) and STSs (14), although with high variability (14). Also, CAFs of canine mammary tumors (15) and corresponding pulmonary metastases (16), pulmonary adenocarcinoma (16), Mast Cell Tumor (MCT) (17), and Soft Tissue Sarcoma (STS) (14) have been found to express FAP.

The FAP-positive CAFs are major producers of extra domain-B fibronectine (EDB+FN) (18).

Fibronectin (FN) is a glycoprotein component of the ECM (19, 20). The FN is synthesized by several cell types (20), including fibroblasts, and has a role in cell adhesion, growth, migration, and differentiation (20). Fibronectin exists in several splice variants (21); one of these variants comprises in its primary sequence a string of 91 amino acids constituting the extra domain-B (EDB) (22), thus named EDB fibronectin (EDB+FN). In humans, EDB+FN is virtually absent in healthy adult tissues, while it is physiologically expressed in tissues during embryonic development, angiogenesis (23), and tissue repair (24, 25).

EDB+FN is expressed by CAFs (19) and endothelial cells of tumor stroma in many human malignancies, including carcinomas (23, 26), adenocarcinomas, sarcomas (26), lymphomas (26, 27), and melanomas (26).

Tumor cells can contribute to the biosynthesis of EDB+FN, as observed in several cancers in human patients, such as head and neck squamous cell carcinoma (26), thyroid and glandular cancers (26), Hodgkin lymphomas (28), mesotheliomas (29) and non-small cell lung carcinomas (29), where EDB+FN has been found expressed not only in stromal cells but also in neoplastic cells. Several studies have reported a positive correlation between EDB+FN expression levels and increased tumor grade and/or aggressiveness (26, 27, 29, 30).

In humans, EDB+FN is a specific marker of angiogenesis, expressed in intratumoral newly formed blood vessels (23, 26), thus representing a potential target for cancer diagnosis and therapy (22, 27, 31–33). EDB+FN targeting drugs have provided great efficacy when combined with other traditional therapies such as checkpoint inhibitors (e.g., PD-1 blockers) (32, 34).

Studies on EDB+FN expression in canine tissues are limited. An EDB+FN mRNA and corresponding protein are present in chondrocytes derived from different normal cartilaginous tissues, except for tracheal cartilage (35). Also, EDB+FN expression in cartilage from canine osteoarthritic joints is up to 10 times higher than in normal joint cartilage (36). However, studies regarding EDB+FN expression and specific immunohistochemical localization in canine tumors are lacking.

Characterizing the expression of FAP and EDB+FN in canine tumors may provide useful insights for future translational studies and support the potential use of antibody-drug conjugates (ADCs) in tumor diagnosis and therapy in veterinary medicine. Thus, this study aims to investigate the FAP and EDB+FN protein expression in selected canine malignant tumors, and to assess their spatial distribution in four tumoral compartments defined as: neoplastic cells, CAFs, cancer associated blood vessels (CAVs) and stromal extracellular matrix.

## Materials

### Ethics

This study was approved by the Institutional Animal Welfare organization called the Animal Welfare Organization (OPBA) of the University of Milano, Italy, and identified by the Protocol #60–2022. Owners signed a written informed consent for all diagnostic and therapeutic procedures and the use of results for scientific purposes.

The work described in this manuscript involved the use of non-experimental (owned) animals bearing spontaneous tumors and subjected to biopsy or surgical excision with diagnostic or curative intent. Established internationally recognized high standards (“best practice”) of veterinary clinical care for the individual patient were always followed.

### Case selection and tissue collection

Paraffin embedded biopsy samples of canine Soft Tissue Sarcoma (STS), Osteosarcoma (OSA), Hemangiosarcoma (HSA), Canine Apocrine Gland Anal Sac Adenocarcinoma (AGASAC), Lymphoma, Mast Cell Tumor (MCT), and Melanoma were retrospectively collected from the electronic archives of the Department of Veterinary Medicine and Animal Sciences (DIVAS) of the University of Milano (Italy) from 2005 to 2022. Retrospectively selected cases were obtained from routinely formalin-fixed and paraffin-embedded (FFPE) tissues collected for diagnostic purposes. Prospective collection of canine spontaneous tumors was instead performed between 2022 and 2024, from dogs admitted to the routine clinical and surgical activities of the Small Animal Veterinary Teaching Hospital of the Department of Veterinary Medicine and Animal Sciences (DIVAS) of the University of Milano (Italy). For prospectively collected cases, neoplastic tissue samples were routinely FFPE and one to multiple small 3×3 mm fragments of fresh neoplastic tissues were snap-frozen via immersion in liquid nitrogen, and embedded in Optimal Cutting Temperature (OCT) compound and stored at −80 °C.

Additionally, normal canine tissue microarrays composed of 15 canine fresh normal tissues collected during necropsies from two dogs deceased of traumatic causes were manufactured to study FAP and EDB+FN expression in normal canine tissues. Details of array construction and the list of tissues included are provided in Supplementary Table 1.

### Histology

For each case included in the study, 4 μm sections were stained with Hematoxylin and Eosin (H&E) and diagnoses were reviewed by four pathologists, one (PR) certified by the European College of Veterinary Pathology (ECVP) board, two ECVP residents (SD, GB), and one doctor of veterinary medicine (DVM, VB) to confirm the diagnosis.

### Markers

Primary antibodies used to assess FAP and EDB+FN expressions in canine tissue were monoclonal and polyclonal markers raised against human molecules and are listed in Table 1.

**Table 1:** List of antibodies used to immunolabel FAP and EDB-Fibronectin.

Antibody	Specificity	Host specie	Dilution and incubation	Retrieval	Secondary antibody	Tertiary antibody
Anti-FAP polyclonal (AbCam ab53066)	Human Fibroblast Activation Protein Alpha^1^	Rabbit	1:200 1 h RT	BufferH, 97 °C, 20 min	Anti-Rabbit Goat IgG 1:200	
Anti-FAP monoclonal (Clone EPR20021, AbCam ab207178)	Human Fibroblast Activation Protein Alpha^1^	Rabbit	1:100 1 h RT	BufferH, 97 °C, 20 min	Anti-Rabbit Goat IgG 1:200	
L19-FITC (Philochem)	EDB Fibronectine	Recombinant	1:100 1 h RT	BufferH, 97 °C, 20 min	Anti-FITC Rabbit IgG 1:1000	Anti-Rabbit in Goat IgG
Anti-Von Willebrand (Dako)	Von Willebrand Factor	Rabbit	Ready to use	BufferH, 97 °C, 20 min	Anti-Rabbit Goat IgG 1:200	

RT, room temperature.

1 The exact immunogen used to generate this antibody is proprietary information.

Briefly, anti-FAP polyclonal primary antibody (ab53066, AbCam, Cambridge, UK), anti-FAP monoclonal primary antibody (clone EPR20021, ab207178, AbCam, Cambridge, UK), or the EDB-targeting L19 recombinant primary antibody (Philogen S.p.A., Siena, IT) conjugated with FITC (L19-FITC).

The cross-reactivity of all three antibodies with canine tissues has been predicted by sequence homology. The three antibodies have also been used in previously published works (14, 17, 37).

### Protein homology

To further support the suitability of human-directed antibodies for the expression of FAP and EDB+FN in canine normal and tumor tissues, aminoacidic sequence alignments between human and canine proteins were performed. Human FAP (UniProt Code: Q12884-1) and canine FAP (UniProt Code: A0A8C0NKP1) sequences were retrieved from the UniProt database (38) and the alignments were conducted using the Clustal Omega (39) algorithm. Similarly the aminoacidic sequence alignment was performed among human EDB+FN (UniProt Code: P02751-7), canine EDB+FN (UniProt Code: Q28275-4), and the EDB epitope recognized by the L19 anti-EDB antibody, which sequence was retrieved from literature (31).

### Immunohistochemistry

The FAP and EDB+FN protein expression was assessed on FFPE samples of tumoral and normal canine tissues, and in frozen tissues from prospectively collected cases after optimization of the immunohistochemical protocols for each specific marker. The antibodies used are listed in Table 1 and protocols are detailed in Supplementary Table 1.

Briefly, FFPE tissues were cut at 4 μm, mounted on poly-L-lysine-coated slides and subjected to combined deparaffinization, hydration and antigen retrieval in alkaline buffer (Buffer H, Epredia, Breda, Netherlands) at 97 °C for 20 min, followed by quenching of endogenous peroxidase and blocking of non-specific binding sites. For FAP, sections were incubated with either polyclonal (1:200) or monoclonal (clone EPR20021, ab207178, AbCam, Cambridge, UK, 1:100) anti-FAP antibodies for 1 h at room temperature, followed by biotinylated secondary antibody. Detection was performed with avidin–biotin complex (ABC kit, Vectastain, Vector, CA, USA), and DAB chromogen (ImmPACT DAB kit, Vector, CA, USA). For EDB+FN, sections were incubated with L19-FITC (Philogen S.p. A., Siena, IT, 1:100) for 1 h, followed by incubation with an anti-FITC antibody and the same detection system. Slides were counterstained with Mayer’s hematoxylin (Diapath, Bergamo, Italy) and mounted.

Frozen canine tumor samples, embedded in Optimal Cutting Temperature (OCT) compound, were cut at 8 μm; sections were mounted on poly-L-lysine-coated slides, fixed in cold acetone and stained with either anti-FAP monoclonal primary antibody, or L19-FITC primary antibody. Signal detection, reaction development and counterstaining were performed as described above.

In each IHC experiment, positive and negative controls were included. Positive controls consisted of a known FAP positive canine sarcoma and three tumor samples derived from the HT-1080 cell line engineered to express murine, human, or canine FAP, previously implanted subcutaneously and grown in murine models (Philochem, Otelfingen, CH). Negative controls consisted of primary antibody replacement with an isotype-matched irrelevant antibody (Rabbit anti-Von Willebrand Factor, Dako, Agilent Technologies, Glostrup, Denmark), omission of the primary antibody, and inclusion in the run of a known FAP negative tumor sample, derived from the HT-1080 wild-type cell line, grown in mouse (Philochem, Otelfingen, CH).

### Immunohistochemistry stain evaluation

Stain distribution was semi-quantitatively evaluated by visually estimating the percentage of neoplastic cells, cancer-associated fibroblasts (CAF), cancer-associated blood vessels (CAV) or tumoral stromal extracellular matrix (ECM) expressing either FAP or EDB+FN, as previously reported (14).

The score applied to evaluate the percentage of positivity was: 1, staining in <1%, considered negative; 2, 1–10%; 3, 11–50%; 4, >50%.

The stain intensity was visually assessed and scored as 0, absent; 1, weak; 2, moderate; 3, strong.

High expression of the marker was defined as expression with moderate to intense (score 2 or 3) intensity in more than 50% (score 4) of the considered compartment, coherently with previously published methods (14, 17).

### Statistical analysis

For each compartment (tumor cells, CAF, CAV and ECM) of every FFPE case, a combined immunoreactivity score was calculated for both the monoclonal and the polyclonal anti-FAP antibodies by multiplying the distribution and intensity scores. Paired comparisons between polyclonal and monoclonal antibodies were performed using the Wilcoxon signed-rank test, for the whole cohort and stratifying by tumor type, for histotypes with more than 10 cases included. In addition, the concordance between polyclonal and monoclonal combined scores within each compartment was assessed using Spearman’s rank correlation coefficient. Statistical analyses were performed using JASP software (Version 0.95.4) (40), and a *p* value < 0.05 was considered statistically significant.

## Results

### Case selection

Retrospective cases included in this study comprised 10 each of the following spontaneous canine malignant tumor types: Hemangiosarcoma (HSA), Osteosarcoma (OSA), cutaneous Soft Tissue Sarcoma (STS), Canine Apocrine Gland Anal Sac Adenocarcinoma (AGASAC), Nodal Lymphoma, Mast Cell Tumor (MCT), and Melanoma (6 oral, 4 cutaneous) for a total of 70 cancers. In addition, 18 prospectively collected cases were included: 5 STS, 8 MCT, 2 melanomas, 2 HSA, and 1 multicentric nodal B-cell lymphoma.

Signalment of dogs, tumor diagnoses, subtypes and grades (when applicable) are summarized in Tables 2 and 3 and detailed in Supplementary Table 2.

**Table 2:** Summary of tumor types and animals’ sex of retrospectively collected cases, for which FFPE samples were available.

Tumor type	Grade/subtype	Number of cases	Male	Female	Unknown	Total
STS	1st	5				
	2nd	2				
	3rd	3				
	Total STS	10	2	5	3	10
OSA		10	5	5	0	10
HSA		10	4	6	0	10
AGASAC		10	4	6	0	10
Nodal lymphomas		10	4	6	0	10
MCT	Grade 1 (low Kiupel)	2				
	Grade 2 (low Kiupel)	2				
	Grade 2 (high Kiupel)	2				
	Grade 3 (high Kiupel)	4				
	Total MCT	10	1	8	1	10
Melanoma		10	4	6	0	10
Total cases		70	24	42	4	70

**Table 3:** Summary of tumor types and animals’ sex of prospectively collected cases, for which both FFPE and frozen samples were available.

**Tumor type**	**Grade**	**Number of cases**	**Male**	**Female**
Mast Cell Tumor	Grade 2 (low Kiupel)	5		
	Grade 3 (high Kiupel)	1		
	Subcutaneous	2		
	Total MCT	8	4	4
Soft Tissue Sarcoma	1st	1		
	2nd	2		
	3rd	2		
	Total STS	5	1	4
Melanoma		2	0	2
Hemangiosarcoma		2	2	0
Nodal lymphomas	High	1	1	0
Total cases		18	8	10

### Protein homology

Based on the aminoacidic sequence alignment, human and canine FAP demonstrated high homology (83.5 homology score), further supporting the use of the anti-human FAP antibodies that were previously used in canine tissues (14, 17).

For the EDB+FN, human and canine proteins demonstrated high homology (83.6 homology score). More importantly, human and canine EDB domains had complete sequence identity with a homology score of 100, guaranteeing cross-reactivity of the human anti-EDB L19 antibody. Furthermore, anti-EDB L19 antibody has also been previously validated for use in canine tissues (37).

### Immunohistochemical expression of FAP and EDB+FN in normal tissue microarray

Assessment of normal canine tissues showed negative FAP or EDB+FN expressions except for epithelial cells in salivary gland ducts and cutaneous apocrine sweat glands that exhibited an intense cytoplasmic labelling.

The expression was also observed in the cytoplasm of stromal fibroblasts of tissues undergoing remodeling/repair (pancreas with fibrosis) or with mild chronic inflammation (kidney with mild interstitial nephritis).

### Immunohistochemical expression of FAP and EDB+FN in canine spontaneous tumors

#### Overall FAP and EDB+FN expression in tumors

FAP expression was observed in all the analysed malignancies in variable percentages of tumors per group, and of cells in each tumor type. Stain intensity differed depending on the primary antibody applied and the tumor type.

The FFPE and frozen samples of the same tumor type (prospective cases) had comparable immunoreactivity, with slightly lower positivity rates in frozen sections, as detailed in Table 4. Nonetheless, in FFPE samples a background stain was often present, especially with polyclonal anti-FAP antibody.

**Table 4:** Expression of FAP and EDB in the four compartments: comparative results in the FFPE and frozen samples.

Tumor type	Cases	**Antibody**	**FFPE**								**FROZEN**							
			TC	%	CAF	%	CAV	%	ST	%	TC	%	CAF	%	CAV	%	ST	%
STS	5	FAP Monoclonal	5	100%	4	80%	4	80%	0	0%	5	100%	4^‡^	80%	1^‡^	20%	3	60%
		EDB+FN	4	80%	3°	60%	3	60%	1	20%	4	80%	3^‡^	60%	3^‡^	60%	1	20%
MCT	8	FAP Monoclonal	5	63%	8	100%	7	88%	0	0%	4	50%	7	88%	1^‡^	13%	1	13%
		EDB+FN	6	75%	2	25%	4	50%	1	13%	8	100%	8	100%	8	100%	4	50%
Melanoma	2	FAP Monoclonal	2	100%	2	100%	2	100%	0	0%	0	0%	2	100%	0	0%	1	50%
		EDB+FN	2	100%	1	50%	2	100%	1	50%	1	50%	2	100%	2	100%	2	100%
HSA	2	FAP Monoclonal	2	100%	2	100%	2	100%	0	0%	1	50%	1	50%	1	50%	0^†^	0%
		EDB+FN	1	50%	1	50%	1	50%	1	50%	1	50%	1	50%	1	50%	1^†^	50%
Lymphoma	1	FAP Monoclonal	1	100%	1	100%	1	100%	0	0%	1	100%	1	100%	1	100%	1	100%
		EDB+FN	0	0%	0	0%	0	0%	0	0%	1	100%	1	100%	1	100%	0	0%
Total	18	FAP Monoclonal	15	83%	17	94%	16	89%	0	0%	11	61%	15	83%	4^‡^	22%	6	33%
		EDB+FN	13	72%	7	39%	10	56%	4	22%	15	83%	15	83%	15^‡^	83%	8	44%

TC, tumoral cells; CAF, cancer associated fibroblasts; CAV, blood vessels; ST, stroma.

‡ Structure not present in all sections.

° Structure not distinguishable in all sections.

† One case was not assessable in frozen sections.

The EDB+FN expression varied in the tumor types, with a vascular staining pattern most commonly observed, and a more frequent expression by CAFs over neoplastic cells. Compared to FAP, EDB+FN expression was more frequent in stroma but less so in CAFs and tumoral cells in FFPE tissues. The EDB+FN labelled a higher percentage of cells, and with higher staining intensity in frozen samples, compared to FFPE.

The FAP and EDB+FN were expressed in the same tumor with a high score (score 4) in 29/88 (33%) cases; of these, 22 cases had at least one compartment expressing both markers. The highest number of double-positive cases was observed in melanomas (8/12, 67%), followed by STS (8/15, 53%), AGASAC (5/10, 50%), and MCT (8/18, 44%). Double expression was less frequent in OSA, and rare in HSA and Lymphoma. In most tumor cases with double expression, the FAP and EDB+FN labelling in multiple compartments was more common than the expression in one compartment, indicating that FAP and EDB+FN are often expressed in different cellular or stromal compartments within the same tumor.

#### FAP and EDB+FN expression in the tumoral compartments

Neoplastic FAP and EDB+FN expressions in FFPE samples are summarized in Table 5 for all spontaneous tumors included in the study.

**Table 5:** Expression of FAP and EDB+FN in the various compartments of the different tumor types.

Tumor type	Cases	Antibody	TC		CAF		CAV		Stroma	
			N°	%	N°	%	N°	%	N°	%
STS	15	FAP Polyclonal	14	93%	13	87%	14	93%	1	7%
		FAP Monoclonal	13	87%	11	73%	11	73%	1	7%
		EDB+FN	10	67%	7	47%	9	60%	5	33%
OSA	10	FAP Polyclonal	9	90%	8	80%	10	100%	0	0%
		FAP Monoclonal	7	70%	8	80%	9	90%	1	10%
		EDB+FN	4	40%	2	20%	3	30%	2	20%
HSA	12	FAP Polyclonal	11	92%	12	100%	12	100%	0	0%
		FAP Monoclonal	6	50%	10	83%	10	83%	1	8%
		EDB+FN	2	17%	4	33%	3	25%	5	42%
AGASAC	10	FAP Polyclonal	10	100%	9	90%	10	100%	2	20%
		FAP Monoclonal	10	100%	9	90%	10	100%	2	20%
		EDB+FN	7	70%	4	40%	8	80%	4	40%
Nodal lymphomas	11	FAP Polyclonal	10	91%	10	91%	11	100%	0	0%
		FAP Monoclonal	6	55%	7	64%	11	100%	0	0%
		EDB+FN	2	18%	1	9%	6	55%	1	9%
MCT	18	FAP Polyclonal	18	100%	18	100%	17	94%	0	0%
		FAP Monoclonal	12	67%	15	83%	10	56%	0	0%
		EDB+FN	6	33%	4	22%	8	44%	3	17%
Melanoma	12	FAP Polyclonal	7	58%	9	75%	8	67%	0	0%
		FAP Monoclonal	6	50%	9	75%	5	42%	0	0%
		EDB+FN	12	100%	9	75%	10	83%	3	25%
TOTAL	88	FAP Polyclonal	79	90%	79	90%	82	93%	3	3%
		FAP Monoclonal	60	68%	69	78%	66	75%	5	6%
		EDB+FN	43	49%	31	35%	47	53%	23	26%

Expression evaluated on FFPE samples. TC, tumoral cells; CAF, cancer associated fibroblasts; CAV, blood vessels.

**Figure 1:**
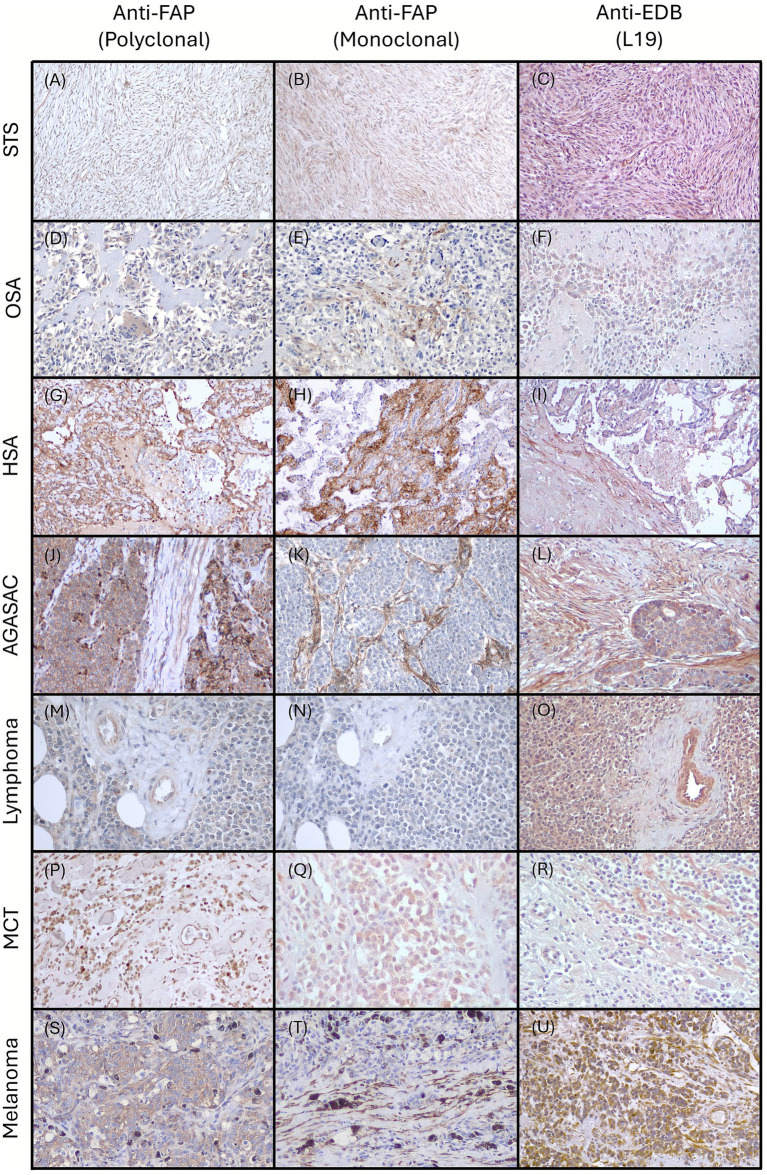
Representative immunohistochemical staining of FAP, detected with the polyclonal and monoclonal antibodies, and EDB+FN, detected with L19 antibody, in canine tumors. **(A–C)** Soft Tissue Sarcoma (STS) (Perivascular Wall Tumor). Neoplastic cells are positively labeled by **(A)** FAP polyclonal, **(B)** FAP monoclonal, and **(C)** L19 antibodies. **(D–F)** Osteosarcoma (OSA). **(D)** Neoplastic cells and multinucleated cells are stained with FAP polyclonal antibody. **(E)** FAP labelling of CAFs with monoclonal antibody. **(F)** Neoplastic cells are EDB+FN positive with L19 antibody. **(G–I)** Hemangiosarcoma (HSA). **(G)** FAP polyclonal antibody labels neoplastic cells and stromal CAFs. **(H)** FAP monoclonal antibody labels neoplastic cells and stromal fibroblasts. Stroma is weakly positive. EDB+FN is expressed by neoplastic cells and CAFs. Stroma is weakly positive. **(J–L)** Apocrine glands anal sac adenocarcinoma (AGASAC). **(J)** CAFs, Vessel endothelium and Neoplastic cells are variably positive with polyclonal anti-FAP antibody. **(K)** anti-FAP monoclonal antibody strongly labels CAFs, while neoplastic cells have a weak staining. **(L)** L19 anti-EDB antibody labels both neoplastic cells and CAFs. **(M–O)** Lymphoma. **(M)** Neoplastic cells and endothelia are moderately positive with FAP polyclonal antibody, and negative to weakly positive with **(N)** FAP monoclonal antibody. **(O)** Neoplastic cells, CAFs, and blood vessel walls strongly express EDB+FN with L19 antibody. **(P–R)** Mast Cell Tumor (MCT). Neoplastic cells and endothelia are labelled by both **(P)** polyclonal and **(Q)** monoclonal anti-FAP antibodies. Stroma is negative. **(R)** L19 antibody labels endothelia and CAFs. **(S–U)** Melanoma. **(S)** Neoplastic cells and CAFs express FAP with polyclonal antibody. **(T)** FAP monoclonal antibody labels CAFs. **(U)** L19 anti-EDB antibody strongly labels neoplastic cells, CAFs, and endothelia.

**Figure 2:**
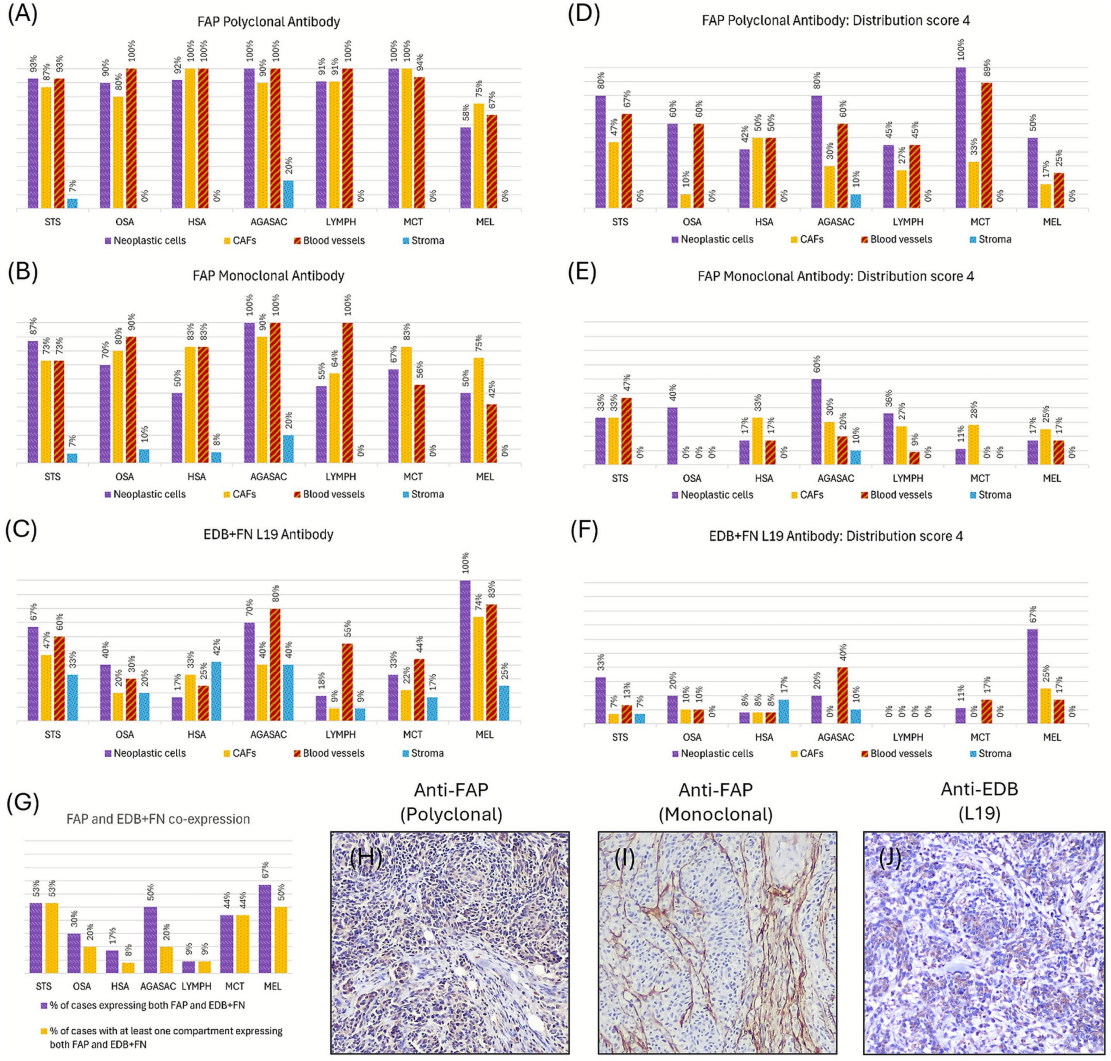
Immunohistochemical evaluation of FAP and EDB+FN expression in canine tumors. **(A–C)** Overall frequency of positive staining (all distribution scores combined) obtained with **(A)** polyclonal anti-FAP antibody, **(B)** monoclonal anti-FAP antibody, and **(C)** L19 anti-EDB+FN antibody across seven tumor types: soft tissue sarcomas (STS), osteosarcomas (OSA), hemangiosarcomas (HSA), anal sac apocrine gland adenocarcinomas (AGASAC), lymphomas (LYMPH), mast cell tumors (MCT), and melanomas (MEL). Staining was assessed in neoplastic cells, cancer-associated fibroblasts (CAFs), tumor-associated blood vessels, and extracellular stroma. **(D–F)** Distribution of the high-expressing cases (distribution score 4) for each antibody and stratified by tumor type and compartment: **(D)** polyclonal anti-FAP, **(E)** monoclonal anti-FAP, and **(F)** L19 anti-EDB+FN. **(G)** Proportion of cases expressing both FAP and EDB+FN regardless of the compartment, and proportion of cases with at least one compartment expressing both FAP and EDB+FN, stratified by tumor type. **(H–J)** Representative immunohistochemical staining of the same canine melanoma demonstrating co-expression of FAP and EDB+FN within the tumor. **(H)** FAP expression with the polyclonal antibody, showing cytoplasmic labeling in neoplastic cells. **(I)** FAP expression with the monoclonal antibody, highlighting tumor-associated fibroblasts. **(J)** EDB+FN expression with the L19 antibody, showing cytoplasmic labeling of neoplastic cells and CAFs. Together, these panels illustrate that a single tumor can concurrently express both FAP and EDB+FN across multiple compartments.

Representative images of FAP and EDB+FN expressions in selected malignancies are depicted in Figure 1. Figure 2 summarizes the results of immunohistochemical expression of the different antibodies in the 4 considered compartments of the various tumor types.

Briefly, expression of FAP and EDB+FN in the different compartments resulted as follows:

#### Neoplastic cell compartment

Neoplastic cells expressed FAP in all tumor types, although with variable frequency and stain intensity according to tumor types.

Neoplastic cells expressed FAP in 79/88 (90%) with the polyclonal antibody and in 60/88 (68%) cases with the monoclonal antibody. In 60 cases the polyclonal antibody labelled more than 50% of the neoplastic cells. The FAP expression in neoplastic cells was cytoplasmic in all tumor types, except in mast cell tumors, where an occasional membrane staining was observed.

Mast cell tumors, AGASAC and STS had strongest FAP labelling with more than 50% of the tumors expressing FAP in more than 50% of neoplastic cells.

The EDB+FN expression in neoplastic cells was observed in 43/88 (49%). Only melanomas consistently expressed EDB+FN in neoplastic cells, with 8/12 (67%) of the cases also having >50% positive cells. Other EDB+FN tumors with neoplastic cell reactivity included STS and AGASAC. OSA and lymphoma had the lowest expression. Detection rates were higher in frozen sections than in FFPE samples from prospective cases, where 15/18 (83%) had positive neoplastic cells.

#### CAF compartment

The FAP expression in CAFs was prominent in all tumor types and more consistent than in neoplastic cells. A total of 79/88 cases (90%) stained positive for CAFs with the polyclonal antibody, and 69/88 (78%) stained with the monoclonal antibody. Expression in frozen tissues was similar. Notably, only in STS more than 50% of cases had FAP expression in more than 50% of CAFs.

The CAF staining was generally more intense than that of tumoral cells and slightly more consistent between FAP antibodies.

The CAFs expressed EDB+FN in 31/88 cases (35%) in FFPE sections and 15/18 (83%) in frozen samples. Across tumor types, CAFs positivity to EDB+FN was unexpectedly low, except for Melanomas and AGASAC. In Melanoma, CAFs labelled for EDB+FN in 9/12 (75%) of the cases, while AGASAC had high CAFs positivity (4/10, 40%), supporting the role of EDB+FN in the desmoplastic stroma of carcinomas.

Overall, CAF positivity for EDB+FN was most robust in melanomas and AGASAC, with excellent preservation in frozen samples. OSA and lymphoma had the lowest expression.

#### Cancer-associated vessel (CAVs) compartment

The CAVs expressed FAP in 82/88 (93%) cases with the polyclonal antibody and in 66/88 (75%) with the monoclonal marker.

The CAVs were consistently FAP-positive in all tumor types except for melanoma, where CAVs expressed FAP in 8/12 (67%) of cases. Due to the limited amount of tissue in the frozen samples, CAVs were available for examination in only 13/18 of the prospective cases. In these, FAP positivity was variable and observed in 4/13 (31%) cases. Cytoplasmic positivity characterized endothelial cells and/or vascular smooth muscle cells (VSMCs) in both FFPE and frozen samples, and across all tumors. The polyclonal antibody labelled more consistently FAP in CAVs compared to the monoclonal antibody.

The EDB+FN was expressed in CAVs in 47/88 (53%) of the FFPE samples, and in 15/16 (94%) of the frozen samples where CAVs were assessable.

Overall, EDB+FN labelling was most frequent in Melanoma, AGASAC, STS, AGASAC, and Lymphomas. Less than 50% of cases of MCT, OSA, and HSA expressed EDB+FN in CAVs.

#### Cancer associated extracellular stroma

In FFPE samples, extracellular stromal FAP was observed in 3/88 (3%) cases with the polyclonal marker and in 5/88 (6%) with the monoclonal marker in FFPE samples. In frozen prospective samples, 6/18 (33%) cases had positive tumor stroma.

Among the tumor types, stromal FAP positivity was highest in AGASAC, lowest in STS, OSA, and HSA, and absent in Lymphoma, MCT and Melanoma.

Extracellular stromal expression of EDB+FN was markedly higher than stromal FAP expression. The EDB+FN labelling was observed in 23/88 (26%) FFPE cases and 8/18 (44%) frozen.

Among the tumor types, stromal EDB+FN positivity was highest in STS, HSA, and AGASAC, lower in MCT and melanoma, and lowest in OSA and Lymphoma.

### Statistical analysis

Paired Wilcoxon analysis showed that in the whole cohort the combined scores obtained with the polyclonal antibody were significantly higher than those obtained with the monoclonal antibody for tumor cells (*p* < 0.001), CAF (*p* = 0.012) and CAV (*p* < 0.001), whereas no significant difference was observed for the ECM (*p* = 0.442). Spearman’s correlation analysis showed a weak but significant concordance between polyclonal and monoclonal scores in tumor cells (ρ = 0.317, *p* = 0.003) and CAF (ρ = 0.242, *p* = 0.023), while no significant correlation was observed for CAV compartment (ρ = 0.115, *p* = 0.284); a weak but significant correlation was detected for the ECM (ρ = 0.215, *p* = 0.044). When stratified by tumor type, the agreement between antibodies varied across compartments and histotypes, with only sporadic statistically significant differences or correlations. Detailed results are reported in Supplementary Table 3.

## Discussion

This work aimed to assess the expression and compartmental distribution of Fibroblast Activation Protein (FAP) and Extra-Domain B Fibronectin (EDB+FN) in normal canine tissues and in selected aggressive spontaneous tumors, using different antibodies and tissue-preservation techniques.

Interest in the specific molecular expression for canine tissues derives from the established relevance of FAP and EDB+FN as useful diagnostic and therapeutic targets in human oncology (22, 41), while data in veterinary medicine remain scarce (14, 15, 17).

Because canine cancers share biological and histological similarities with their human counterparts (42, 43), the current findings on FAP and EDB+FN expression in canine tumors may further support the translational relevance of the dog as a comparative oncology model, and the applicability of drugs approved for use in human oncology, for canine cancer treatment.

FAP expression assessed by polyclonal and a monoclonal antibodies showed marked differences in staining pattern. The monoclonal antibody produced intense but mainly CAF-restricted labeling, whereas the polyclonal antibody yielded broader and more heterogeneous FAP staining across neoplastic cells, and stromal and vascular compartments.

The FAP expression was predominantly cytoplasmic, except for occasional membranous signal in neoplastic mast cells. Cytoplasmic FAP has also been widely reported in human neoplastic cells (2), and may reflect increased intracellular synthesis, altered dimerization with cytoplasmic retention (44, 45), the presence of an intracellular splice variants (46), or mutations in the transmembrane domain (45). Noteworthy, only membrane-exposed or extracellular FAP and EDB+FN are accessible to targeted therapies, whereas intracellular isoforms, while detectable by immunohistochemistry, do not represent a practical target.

The EDB+FN labeling was also cytoplasmic, with either a diffuse signal or a punctate pattern within the cytoplasm or along its periphery. Although EDB+FN is an extracellular matrix protein, its intracellular detection likely reflects active synthesis and vesicular trafficking prior to deposition, confirming tumoral and vascular cells as its source, as observed in human tumors (47, 48), where it is produced by FAP-positive CAFs, VSMCs, and neoplastic cells during angiogenesis and epithelial–mesenchymal transition (21, 22, 25).

High FAP expression by neoplastic cell was detected in AGASAC with both antibodies, and in over half of MCT, STS, and OSA with the polyclonal antibody, whereas less than 50% of HSA and lymphomas were highly positive.

FAP expression was weak in canine melanoma cells paralleling the generally low expression reported in human melanoma (49). In this tumor type, FAP is physiologically silenced and exert tumor-suppressive effects when re-expressed (50); a mechanism that could occur also in canine melanoma. FAP expression by cells of other human malignancies has been correlated with aggressive biological behavior, including lymphovascular invasion and metastasis (6).

In contrast, EDB+FN expression in neoplastic cells was consistently detected in all canine melanomas, while it was lower and more variable in other tumors, and present mostly in STS and AGASAC. The EDB+FN can be present in neoplastic cells (26–28, 51) and is linked to enhanced adhesion, motility, invasion, EMT, and metastatic potential (26, 27, 30, 52).

An association between FAP or EDB+FN expression and prognosis was beyond the scope of this work, and should be addressed in future investigations.

CAFs had high FAP labelling in HSA, OSA, STS, AGASAC, and MCT, while they consistently expressed EDB+FN only in melanomas.

Because FAP-positive CAFs are major producers of EDB+FN (18) a level of positivity is expected; however, the limited concurrent expression of FAP and EDB+FN in CAFs of our caseload may reflect the dynamic nature of EDB+FN deposition, known to peak in active stromal remodeling, and to decline during matrix maturation, with possible preferential localization at the invasive fronts or in early tumor development (53, 54).

Both FAP and EDB+FN were expressed by endothelial cells and/or VSMCs in all tumor types, in agreement with their known role in angiogenesis and vascular remodeling (2, 26, 51). In human oncology, FAP and EDB+FN are expressed by tumor-associated vascular endothelium, mural cells, and basal membranes, especially at the invasive front (41). The detection of both targets in tumor vessels supports the potential use of targeted anti-angiogenic therapeutic strategies (55, 56) and imaging tracers for early metastasis detection also in dogs.

Tumor stromal ECM had limited or no FAP expression in most tumors, with low-level positivity detected with the monoclonal antibody only in some AGASACs. Contrarily, EDB+FN labelled stromal ECM particularly in HSA, STS and AGASAC, while melanomas had limited EDB+FN stromal expression despite high expression in other compartments. A similar pattern has been described for human melanomas, where stromal EDB+FN is mainly detected in metastatic lesions (57).

In humans, extracellular matrix expression of FAP is rarely overtly reported (58), and the term “stromal positivity” is often used to refer to CAFs, thus hampering parallels. However, FAP has been demonstrated in collagen-rich peritumoral matrix in cerebral metastases of human STS (58). When reported (58), ECM expression is described as of variable intensity, paralleling what observed in our study. Soluble FAP in ECM is likely composed of the FAP extracellular domain (59) shed by CAFs’ invadopodia (60), a mechanism also described in humans’ myocardial infarction (61), and fibrosis (44).

Both FAP and EDB+FN intervene to remodel tumor ECM. FAP, collagenolytic activity (62), promotes fibronectin deposition, including EDB+FN, which dimerize in a specific head-to-tail pattern (63), that expose binding sites for cellular integrins (63), alter collagen alignment, increase matrix stiffness (62, 63), and favor tumor cell adhesion, motility and neoangiogenesis (51, 62, 64). Together, these mechanisms, promotes a desmoplastic microenvironment permissive to tumor invasion (62, 64).

Given the results of this work, desmoplasia and tumor invasion may also be favored in those tumors expressing one or both molecules in the stroma.

Tumor grading was recorded for all cases; however, the limited number of cases per grade within each tumor category precluded robust grade-stratified statistical analyses. Descriptively, no clear grade-dependent trends were observed. While a possible influence of grade on FAP and EDB+FN expression cannot be excluded, this issue will require evaluation in larger cohorts.

A relevant number of canine tumors expressed FAP and EDB+FN concurrently, either in the same or in different compartments, supporting the rationale for dual-targeted therapeutic approaches, particularly in aggressive tumors such as Melanomas and AGASACs.

In murine xenograft models, combined therapy consisting of a FAP-directed therapeutic molecule along with an EDB+FN-directed immunocytokine, achieved complete tumor remission, whereas single-agent treatment produced only partial response (65).

This synergy was attributed to enhanced NK-cell cytotoxicity and simultaneous targeting of both tumoral cells and tumor microenvironment, with disruption of the stromal remodeling and of the tumor vasculature development (65).

The frequent spatial separation of FAP and EDB+FN observed in canine tumors suggests that a combined targeting approach may improve therapeutic efficacy, overcoming the limitations associated with single-target spatial restriction.

Overall, these findings reinforce previous evidence identifying each marker as a potential target for therapy (5, 51), and provide further biological support for dual-agent strategies.

Comparison of the two anti-FAP antibodies showed consistently higher combined scores with the polyclonal antibody across all compartments, whereas the monoclonal antibody produced more restricted but intense CAF labeling.

The only partial concordance in case ranking, particularly for the vascular compartment, suggests that while the polyclonal antibody is generally more sensitive, the two antibodies do not fully overlap in their detection pattern across individual cases. This behavior is compatible with broader epitope recognition by the polyclonal antibody, potentially including additional FAP isoforms or conformational states not efficiently recognised by the monoclonal antibody clone. The sporadic statistically significant differences within individual tumor types most likely reflects the limited statistical power of subgroup analyses, due to limited sample size, and should therefore be interpreted as exploratory.

This difference may reflect the presence of multiple splice variants of FAP (46, 66); while the polyclonal antibody may be able to detect more isoforms, the monoclonal produces a more specific but limited staining, restricted to one isoform that may not always be present. For clinical applications requiring accurate patient selection, the combined use of both antibodies may allow a more accurate identification of FAP expressing tumors.

Based on our work, tissue preservation influenced immunoreactivity similarly to other studies (67). FFPE samples showed excellent morphological preservation and good immunoreactivity for both FAP and EDB+FN, but required prolonged antigen retrieval and frequently exhibited higher background staining, especially with the polyclonal antibody. Conversely, frozen samples showed improved preservation of EDB+FN epitopes, likely reflecting its sensitivity to fixation, and stronger monoclonal FAP labelling, but had reduced architectural preservation, and the limited sample size available for freezing restricted evaluation of all compartments.

This study represents, to the authors’ knowledge, the first comprehensive compartment-specific evaluation of FAP and EDB+FN labelling across multiple aggressive canine tumor types. Limitations of this study include the retrospective nature of some samples, leading to variability in fixation protocols, possibly influencing the staining. Finally, intrinsic differences between FFPE and frozen samples influenced interpretation. Despite these limitations, the consistent detection of FAP and EDB+FN in neoplastic, stromal, and vascular compartments supports their biological relevance in the canine tumor microenvironment and provides a strong rationale for future translational studies exploring their diagnostic, prognostic, and therapeutic potential in veterinary oncology.

## Conclusion

The detection of FAP in neoplastic cells and CAFs and of EDB+FN in tumor stroma supports the potential applicability of FAP- and EDB-targeted strategies in veterinary oncology, for both diagnostic and therapeutic purposes.

Among the tumor types investigated, STS, AGASAC, and MCT most consistently expressed FAP, while oral and cutaneous melanomas showed the highest EDB+FN expression, identifying these tumors as priority candidates for future targeted drug studies. For patient selection in future studies of FAP-targeted therapies, employing a combined assessment using both monoclonal and polyclonal antibodies may enhance the detection of potential FAP isoforms and thereby ensure more accurate enrolment.

Finally, given the documented synergistic efficacy of dual FAP–EDB targeting reported in human oncology, REF the present findings indicate that a subset of canine patients, particularly those affected by melanoma and AGASAC, may benefit from comparable therapeutic strategies.

## Data Availability

The original contributions presented in the study are included in the article/Supplementary material, further inquiries can be directed to the corresponding author.
