# Supplementary material for: Expression patterns of fibroblast activation protein and extra-domain B fibronectin in canine malignant tumors
Source: Front Vet Sci. 2026 Feb 2;12:1719994. doi: 10.3389/fvets.2025.1719994 (PMC12908591; doi:10.3389/fvets.2025.1719994)
Supplement: Supplementary file 1 [file Supplementary_file_1.docx]

**Supplemental Material S1**

**Material and methods**

**Tissue Array production**

A canine tissue microarray (TMA) was constructed to serve as a control for immunohistochemistry. Organs were collected at necropsy from two dogs, one male mixed breed, 5 years old, and one male Romagna Water Dog, 8 years old.
Samples included kidney, liver, lung, heart, urinary bladder, prostate, thyroid, adrenal gland, fat, duodenum, pancreas, spleen, lymph node, salivary gland, and skin. Tissues were routinely processed for paraffin embedding. Hematoxylin and eosin-stained slides were examined microscopically, and representative areas of morphologically preserved tissue were selected. From the corresponding donor blocks, 3-mm cylindrical cores were obtained using a manual punch. A custom silicone mold was used to prepare a recipient paraffin block containing pre-formed holes, into which the tissue cores were inserted. The block was heated in a warm bath and 50°C to ensure integration of the cores with the surrounding paraffin. Serial sections (4 µm) were then cut from the final array block for subsequent immunohistochemical analysis.

**Immunohistochemistry protocols**

**FFPE samples**

Paraffin embedded tissues were cut at 4 μm, mounted on poly-L-lysine-coated slides, and stained with either anti-FAP polyclonal primary antibody (ab53066, AbCam, Cambridge, UK), or anti-FAP monoclonal primary antibody (clone EPR20021, ab207178, AbCam, Cambridge, UK), or L19-FITC recombinant primary antibody (Philogen S.p.A., Siena, IT), whose cross-reactivity with canine tissues has been previously demonstrated.^1–3^

The sections underwent combined deparaffinization, hydration and antigen retrieval in a preheated pH 9 working solution (Buffer H, Epredia, Breda, Netherlands) at 97°C for 20 minutes, followed by endogenous peroxidase activity quenching with 0,3% hydrogen peroxide, aspecific binding site blocking with normal goat serum, and incubation with anti-FAP polyclonal or monoclonal primary antibody (1:200 and 1:100 respectively), or L19-FITC (1:100) antibody for 1 hour at room temperature. L19-FITC staining was followed by incubation with anti-FITC antibody (1:1000) for 30 minutes. After incubation with biotinylated antibody (Goat anti-rabbit, Vector, CA, USA) for 30 minutes at room temperature, detection was performed with the avidin-biotin enzyme complex (ABC kit, Vectastain, Vector, CA, USA) for 30 minutes. The reaction was developed with diaminobenzidine substrate kit (ImmPACT DAB kit, Vector, CA, USA). Sections were counterstained with Mayer’s hematoxylin (Diapath, Bergamo, Italy), dehydrated and mounted with Micromount (Diapath, Bergamo, Italy). Positive controls consisted of the inclusion in the run of known FAP positive canine sarcoma and a known FAP positive murine tumor. Negative controls consisted of primary antibody replacement with an isotype-matched irrelevant antibody (Rabbit anti-Von Willebrand Factor, Dako, Agilent Technologies, Glostrup, Denmark), omission of the primary antibody, and inclusion in the run of a known FAP negative murine tumor.

**Frozen samples**

Fresh frozen canine tumor were embedded in Optimal Cutting Temperature (OCT) compound and 8 μm sections were mounted on poly-L-lysine-coated slides. Sections were brought to room temperature (RT), fixed for 10 minutes in cold acetone and rinsed in PBS. A 30 minutes incubation with normal goat serum was followed by incubation with anti-FAP monoclonal primary antibody (1:100), or L19-FITC (1:100) antibody, 1 hour at room temperature. L19-FITC only was followed by incubation with anti-FITC antibody (1:1000) for 30 minutes. Endogenous peroxidase activity was quenched with 0,3% hydrogen peroxide in methanol. After incubation with biotinylated antibody (Goat anti-rabbit, Vector, CA, USA) for 30 minutes at room temperature, signal detection, reaction development and counterstaining were performed as described above. Positive controls consisted of the inclusion in the run of known FAP positive canine sarcoma and a known FAP positive murine tumors. Negative controls consisted of primary antibody replacement with an isotype-matched irrelevant antibody (Rabbit anti-Von Willebrand Factor, Dako, Agilent Technologies, Glostrup, Denmark), omission of the primary antibody, and inclusion in the run of a known FAP negative murine tumor.

1. Beer P, Pauli C, Haberecker M, et al. Cross-species evaluation of fibroblast activation protein alpha as potential imaging target for soft tissue sarcoma: a comparative immunohistochemical study in humans, dogs, and cats. *Front Oncol*. 2023;**13**:1210004.

2. Giuliano A, dos Santos Horta R, Constantino-Casas F, Hoather T, Dobson J. Expression of Fibroblast Activating Protein and Correlation with Histological Grade, Mitotic Index and Ki67 Expression in Canine Mast Cell Tumours. *J Comp Pathol*. 2017;**156**(1):14–20.

3. Philipp Probst. Antibody-based delivery of TNF for cancer immunotherapy. ETH Zurich 2018:
