# Supplementary material for: Expression patterns of fibroblast activation protein and extra-domain B fibronectin in canine malignant tumors
Source: Front Vet Sci. 2026 Feb 2;12:1719994. doi: 10.3389/fvets.2025.1719994 (PMC12908591; doi:10.3389/fvets.2025.1719994)
Supplement: Supplementary file 2 [file Supplementary_file_2.docx]

**Supplemental Material 2: Statistical Analisis**

**Paired samples Wilcoxon T-test:** comparing combined scores of polyclonal and monoclonal anti-FAP antibody in tumoral cells (TC), Cancer Associated Fibroblasts (CAF), Tumor Vasculature (CAV) and Extracellular Matrix (ECM) in the whole cohort of Formalyn-Fixed Paraffin-Embedded (FFPE) samples (n=88)

|  |  |  |  | 95% CI for Rank-Biserial Correlation | |
| --- | --- | --- | --- | --- | --- |
| Compartment | z | p | Rank-Biserial  Correlation | Lower | Upper |
| TC | 6.353 | < .001*** | 0.874 | 0.793 | 0.924 |
| CAF | 2.506 | .012* | 0.363 | 0.096 | 0.581 |
| CAV | 5.860 | < .001*** | 0.818 | 0.704 | 0.890 |
| ECM | -0.845 | .442 | -0.357 | -0.834 | 0.426 |

* p < .05, ** p < .01, *** p < .001

**Spearman’s correlation:** correlation between combines scores of polyclonal and monoclonal anti-FAP antibody in tumoral cells (TC), Cancer Associated Fibroblasts (CAF), Tumor Vasculature (CAV) and Extracellular Matrix (ECM) in the whole cohort of Formalyn-Fixed Paraffin-Embedded (FFPE) samples (n=88)

| Compartment | Spearman's rho | p |
| --- | --- | --- |
| TC | 0.317 | .003** |
| CAF | 0.242 | .023* |
| CAV | 0.115 | .284 |
| ECM | 0.215 | .044* |

* p < .05, ** p < .01, *** p < .001

**Soft Tissue Sarcoma (STS)**

**Paired samples Wilcoxon T-test:** comparing combined scores of polyclonal and monoclonal anti-FAP antibody in TC, CAF, CAV, and ECM in STS FFPE samples (n=15)

|  |  |  |  | 95% CI for Rank-Biserial Correlation | |
| --- | --- | --- | --- | --- | --- |
| Compartment | z | p | Rank-Biserial  Correlation | Lower | Upper |
| TC | 2.706 | .007** | 0.885 | 0.639 | 0.967 |
| CAF | 1.836 | .074 | 0.689 | 0.110 | 0.919 |
| CAV | 1.608 | .115 | 0.526 | -0.056 | 0.841 |
| ECM | 0.447 | 1.000 | 0.333 | -0.806 | 0.948 |

* p < .05, ** p < .01, *** p < .001

**Spearman’s correlation:** correlation between combines scores of polyclonal and monoclonal anti-FAP antibody in TC, CAF, CAV, and ECM in FFPE samples of STS (n=15)

| Compartment | Spearman's rho | p |
| --- | --- | --- |
| TC | 0.172 | .539 |
| CAF | 0.241 | .386 |
| CAV | -0.302 | .274 |
| ECM | -0.071 | .800 |

* p < .05, ** p < .01, *** p < .001

**Osteosarcoma**

**Paired samples Wilcoxon T-test:** comparing combined scores of polyclonal and monoclonal anti-FAP antibody in TC, CAF, CAV, and ECM in Osteosarcoma FFPE samples (n=10)

|  |  |  |  | 95% CI for Rank-Biserial Correlation | |
| --- | --- | --- | --- | --- | --- |
| Compartment | z | p | Rank-Biserial  Correlation | Lower | Upper |
| TC | 1.750 | .091 | 0.694 | 0.079 | 0.927 |
| CAF | 1.468 | .171 | 0.667 | -0.085 | 0.935 |
| CAV | 2.666 | .008** | 1.000 | 1.000 | 1.000 |
| ECM | NaNᵃ |  |  |  |  |

* p < .05, ** p < .01, *** p < .001
NaN: The variance in ECM is equal to 0

**Spearman’s correlation:** correlation between combines scores of polyclonal and monoclonal anti-FAP antibody in TC, CAF, CAV, and ECM in FFPE samples of Osteosarcoma (n=10)

| Compartment | Spearman's rho | p |
| --- | --- | --- |
| TC | -0.186 | .608 |
| CAF | 0.134 | .713 |
| CAV | 0.510 | .132 |

* p < .05, ** p < .01, *** p < .001

**Hemangiosarcoma**

**Paired samples Wilcoxon T-test:** comparing combined scores of polyclonal and monoclonal anti-FAP antibody in TC, CAF, CAV, and ECM in Hemangiosarcoma FFPE samples (n=12)

|  |  |  |  | 95% CI for Rank-Biserial Correlation | |
| --- | --- | --- | --- | --- | --- |
| Compartment | z | p | Rank-Biserial  Correlation | Lower | Upper |
| TC | 1.718 | .096 | 0.644 | 0.030 | 0.905 |
| CAF | 1.362 | .191 | 0.511 | -0.169 | 0.862 |
| CAV | 1.019 | .331 | 0.364 | -0.308 | 0.793 |
| ECM | Na |  |  |  |  |

* p < .05, ** p < .01, *** p < .001
Na: The variance in ECM is equal to 0

**Spearman’s correlation:** correlation between combines scores of polyclonal and monoclonal anti-FAP antibody in TC, CAF, CAV, and ECM in FFPE samples of Hemangiosarcoma (n=12)

| Compartment | Spearman's rho | p |
| --- | --- | --- |
| TC | 0.081 | .802 |
| CAF | 0.083 | .797 |
| CAV | 0.208 | .517 |

* p < .05, ** p < .01, *** p < .001

**Apocrine Gland Anal Sac Adenocarcinoma (AGASAC)**

**Paired samples Wilcoxon T-test:** comparing combined scores of polyclonal and monoclonal anti-FAP antibody in TC, CAF, CAV, and ECM in AGASAC FFPE samples (n=10)

|  |  |  |  | 95% CI for Rank-Biserial Correlation | |
| --- | --- | --- | --- | --- | --- |
| Compartment | z | p | Rank-Biserial  Correlation | Lower | Upper |
| TC | 2.192 | .032* | 0.822 | 0.404 | 0.956 |
| CAF | 0.070 | 1.000 | 0.028 | -0.635 | 0.667 |
| CAV | 1.775 | .088 | 0.750 | 0.144 | 0.947 |
| ECM | 0.000 | 1.000 | 0.000 | -0.840 | 0.840 |

* p < .05, ** p < .01, *** p < .001

**Spearman’s correlation:** correlation between combines scores of polyclonal and monoclonal anti-FAP antibody in TC, CAF, CAV, and ECM in FFPE samples of AGASAC (n=10)

| Compartment | Spearman's rho | p |
| --- | --- | --- |
| TC | 0.118 | .746 |
| CAF | 0.344 | .330 |
| CAV | 0.320 | .367 |
| ECM | 0.364 | .301 |

* p < .05, ** p < .01, *** p < .001

**Lymphoma**

**Paired samples Wilcoxon T-test:** comparing combined scores of polyclonal and monoclonal anti-FAP antibody in TC, CAF, CAV, and ECM in Limphoma FFPE samples (n=11)

|  |  |  |  | 95% CI for Rank-Biserial Correlation | |
| --- | --- | --- | --- | --- | --- |
| Compartment | z | p | Rank-Biserial  Correlation | Lower | Upper |
| TC | 2.666 | .007** | 1.000 | 1.000 | 1.000 |
| CAF | 2.201 | .034* | 1.000 | 1.000 | 1.000 |
| CAV | 2.521 | .014* | 1.000 | 1.000 | 1.000 |
| ECM | Na |  |  |  |  |

* p < .05, ** p < .01, *** p < .001
Na: The variance in ECM is equal to 0

**Spearman’s correlation:** correlation between combines scores of polyclonal and monoclonal anti-FAP antibody in TC, CAF, CAV, and ECM in FFPE samples of Lymphoma (n=11)

| Compartment | Spearman's rho | p |
| --- | --- | --- |
| TC | 0.875 | < .001*** |
| CAF | 0.860 | < .001*** |
| CAV | 0.132 | .699 |

* p < .05, ** p < .01, *** p < .001

**Mast Cell Tumors (MCT)**

**Paired samples Wilcoxon T-test:** comparing combined scores of polyclonal and monoclonal anti-FAP antibody in TC, CAF, CAV, and ECM in MCT FFPE samples (n=18)

|  |  |  |  | 95% CI for Rank-Biserial Correlation | |
| --- | --- | --- | --- | --- | --- |
| Compartment | z | p | Rank-Biserial  Correlation | Lower | Upper |
| TC | 3.724 | < .001*** | 1.000 | 1.000 | 1.000 |
| CAF | 1.420 | .161 | 0.392 | -0.126 | 0.742 |
| CAV | 3.621 | < .001*** | 1.000 | 1.000 | 1.000 |
| ECM | Na |  |  |  |  |

* p < .05, ** p < .01, *** p < .001
Na: The variance in ECM is equal to 0

**Spearman’s correlation:** correlation between combines scores of polyclonal and monoclonal anti-FAP antibody in TC, CAF, CAV, and ECM in FFPE samples of MCT (n=18)

| Compartment | Spearman's rho | p |
| --- | --- | --- |
| TC | -0.143 | .571 |
| CAF | -0.198 | .430 |
| CAV | -0.003 | .991 |

* p < .05, ** p < .01, *** p < .001

**Melanoma**

**Paired samples Wilcoxon T-test:** comparing combined scores of polyclonal and monoclonal anti-FAP antibody in TC, CAF, CAV, and ECM in Melanoma FFPE samples (n=12)

|  |  |  |  | 95% CI for Rank-Biserial Correlation | |
| --- | --- | --- | --- | --- | --- |
| Compartment | z | p | Rank-Biserial  Correlation | Lower | Upper |
| TC | 1.483 | .176 | 0.733 | -0.033 | 0.957 |
| CAF | -0.350 | .778 | -0.139 | -0.725 | 0.563 |
| CAV | 2.023 | .058 | 1.000 | 1.000 | 1.000 |
| ECM | Na |  |  |  |  |

* p < .05, ** p < .01, *** p < .001
Na: The variance in ECM is equal to 0

**Spearman’s correlation:** correlation between combines scores of polyclonal and monoclonal anti-FAP antibody in TC, CAF, CAV, and ECM in FFPE samples of Melanoma (n=12)

| Compartment | Spearman's rho | p |
| --- | --- | --- |
| TC | 0.540 | .070 |
| CAF | 0.110 | .733 |
| CAV | 0.524 | .080 |

* p < .05, ** p < .01, *** p < .001
