# Supplementary material for: Expression patterns of fibroblast activation protein and extra-domain B fibronectin in canine malignant tumors
Source: Front Vet Sci. 2026 Feb 2;12:1719994. doi: 10.3389/fvets.2025.1719994 (PMC12908591; doi:10.3389/fvets.2025.1719994)
Supplement: Supplementary file 3 [file Table_1.docx]

**Supplemental Table 1:** Retrospectively and prospectively collected cases: signalment and tumour type and grade.

| Case N° | R/P | Breed | Sex | Age | Tumour | Subtype / grade |
| --- | --- | --- | --- | --- | --- | --- |
| 1 | R | Golden retriever | FS | 8 | Hemangiosarcoma |  |
| 2 | R | Boxer | F | 13 | Hemangiosarcoma |  |
| 3 | R | Golden retriever | M | 10 | Hemangiosarcoma |  |
| 4 | R | Mixed breed | F | 12 | Hemangiosarcoma |  |
| 5 | R | Mixed breed | M | 13 | Hemangiosarcoma |  |
| 6 | R | Amstaff | F | 8 | Hemangiosarcoma |  |
| 7 | R | Mixed breed | M | 12 | Hemangiosarcoma |  |
| 8 | R | Golden retriever | FS | 10 | Hemangiosarcoma |  |
| 9 | R | Mixed breed | FS | 13 | Hemangiosarcoma |  |
| 10 | R | Dachshund | MC | 5 | Hemangiosarcoma |  |
| 11 | P | Golden retriever | M | 10 | Hemangiosarcoma |  |
| 12 | P | Mixed breed | M | 8 | Hemangiosarcoma |  |
| 13 | R | Leonberger | F | 4 | Osteosarcoma |  |
| 14 | R | Mixed breed | FS | 11 | Osteosarcoma |  |
| 15 | R | Mixed breed | FS | 12 | Osteosarcoma |  |
| 16 | R | Pit bull terrier | M | 10 | Osteosarcoma |  |
| 17 | R | Pit bull terrier | M | 3 | Osteosarcoma |  |
| 18 | R | Poodle | M | 9 | Osteosarcoma |  |
| 19 | R | Labrador retriever | FS | 9 | Osteosarcoma |  |
| 20 | R | Italian cane Corso | M | 11 | Osteosarcoma |  |
| 21 | R | French bulldog | M | 10 | Osteosarcoma |  |
| 22 | R | St. Bernard | M | 14 | Osteosarcoma |  |
| 23 | R | Border collie | M | 4 | Lymphoma | B-cell, Low grade |
| 24 | R | Mixed breed | F | 8 | Lymphoma | T-cell, Low grade |
| 25 | R | Labrador retriever | FS | 8 | Lymphoma | B-cell, Intermediate grade |
| 26 | R | Mixed breed | FS | 15 | Lymphoma | T-cell, Intermediate grade |
| 27 | R | Italin Cane Corso | F | 7 | Lymphoma | T-cell, Low grade |
| 28 | R | Mixed breed | M | 11 | Lymphoma | B-cell, Low grade |
| 29 | R | Bull Terrier | F | 6 | Lymphoma | B-cell, High grade |
| 30 | R | German shepherd | M | 4 | Lymphoma | B-cell, Intermediate grade |
| 31 | R | WHWT | M | 7 | Lymphoma | B-cell, High grade |
| 32 | R | Beagle | FS | 9 | Lymphoma | B-cell, High grade |
| 33 | P | Mixed breed | MC | 5 | Lymphoma | B-cell, High grade |
| 34 | R | Jack Russell terrier | M | 10 | AGASAC |  |
| 35 | R | Cocker Spaniel | F | 10 | AGASAC |  |
| 36 | R | NA | FS | 11 | AGASAC |  |
| 37 | R | Golden retriever | FS | 8 | AGASAC |  |
| 38 | R | Mixed breed | FS | 11 | AGASAC |  |
| 39 | R | Dachshund | M | 13 | AGASAC |  |
| 40 | R | Labrador retriever | FS | 12 | AGASAC |  |
| 41 | R | Border collie | M | 10 | AGASAC |  |
| 42 | R | Hungarian vizsla | FS | 10 | AGASAC |  |
| 43 | R | Irish setter | M | 11 | AGASAC |  |
| 44 | R | German shepherd | F | 7 | STS | Fibrosarcoma, grade I |
| 45 | R | Schnauzer | M | 1 | STS | Fibromixosarcoma, grade I |
| 46 | R | Mixed breed | FS | 9 | STS | Nerve sheath tumour, grade I |
| 47 | R | Dachshund | FS | 15 | STS | PWT, grade III |
| 48 | R | Golden retriever | FS | 13 | STS | Leiomyosarcoma, grade II |
| 49 | R | Mixed breed | M | 15 | STS | Leiomyosarcoma, grade III |
| 50 | R | NA | NA | NA | STS | Rhabdomyosarcoma, grade I |
| 51 | R | English Setter | F | 1 | STS | Rhabdomyosarcoma, grade I |
| 52 | R | Mixed breed | NA | NA | STS | Rhabdomyosarcoma, grade III |
| 53 | R | NA | NA | NA | STS | PWT, grade II |
| 54 | P | Golden retriever | FS | 10 | STS | Undifferentiated, grade III |
| 55 | P | Mixed breed | FS | 11 | STS | Fibromixosarcoma, grade I |
| 56 | P | Mixed breed | M | 13 | STS | Undifferentiated, grade III |
| 57 | P | Mixed breed | FS | 14 | STS | PWT, grade II |
| 58 | P | Mixed breed | F | 13 | STS | PWT, grade II |
| 59 | R | Labrador retriever | F | 6 | Mast Cell Tumour | Patnaik III – High Kiupel |
| 60 | R | Pinscher | FS | 7 | Mast Cell Tumour | Patnaik II – High Kiupel |
| 61 | R | English Setter | FS | 8 | Mast Cell Tumour | Patnaik I – Low Kiupel |
| 62 | R | Mixed breed | FS | 14 | Mast Cell Tumour | Patnaik II – High Kiupel |
| 63 | R | Boxer | FS | 11 | Mast Cell Tumour | Patnaik III – High Kiupel |
| 64 | R | Boxer | NA | 5 | Mast Cell Tumour | Patnaik III – High Kiupel |
| 65 | R | English Setter | FS | 12 | Mast Cell Tumour | Patnaik I/II – Low Kiupel |
| 66 | R | Mixed breed | F | 10 | Mast Cell Tumour | Patnaik II – Low Kiupel |
| 67 | R | Mixed breed | F | 10 | Mast Cell Tumour | Patnaik II – Low Kiupel |
| 68 | R | Boston terrier | M | NA | Mast Cell Tumour | Patnaik III – High Kiupel |
| 69 | P | Golden retriever | MC | 8 | Mast Cell Tumour | Patnaik II – Low Kiupel |
| 70 | P | Italian sighthound | M | 9 | Mast Cell Tumour | Patnaik III – High Kiupel |
| 71 | P | German shepherd | MC | 13 | Mast Cell Tumour | Patnaik II – Low Kiupel |
| 72 | P | Mixed breed | F | 13 | Mast Cell Tumour | subcutaneous |
| 73 | P | English Setter | M | 6 | Mast Cell Tumour | Patnaik II – Low Kiupel |
| 74 | P | Boxer | FS | 4 | Mast Cell Tumour | subcutaneous |
| 75 | P | Golden retriever | FS | 9 | Mast Cell Tumour | Patnaik II – Low Kiupel |
| 76 | P | Mixed breed | FS | 10 | Mast Cell Tumour | Patnaik II – Low Kiupel |
| 77 | R | German shepherd | M | ND | Melanoma | Skin |
| 78 | R | Scottish terrier | M | 10 | Melanoma | Skin |
| 79 | R | Spanish greyhound | M | 10 | Melanoma | Oral |
| 80 | R | French bulldog | FS | 11 | Melanoma | Skin |
| 81 | R | Mixed breed | FS | 11 | Melanoma | Oral |
| 82 | R | Golden retriever | FS | 11 | Melanoma | Oral |
| 83 | R | Jack Russell terrier | M | 11 | Melanoma | Oral |
| 84 | R | Mixed breed | FS | 7 | Melanoma | Skin |
| 85 | R | Mixed breed | FS | 12 | Melanoma | Oral |
| 86 | R | Mixed breed | FS | 12 | Melanoma | Oral |
| 87 | P | Mixed breed | FS | 11 | Melanoma | Oral |
| 88 | P | Golden retriever | F | 11 | Melanoma | Oral |

Sympbols: R/P: Retrospective/Prospective; F: Female; FS: Spayed Female; M: Male; MC: Castrated Male; Amstaff: American Staffordshire terrier; WHWT: West highland white terrier; STS: Soft tissue sarcoma; PWT: Perivascular wall tumour; AGASAC: Apocrine gland anal sac adenocarcinoma; NA: not available
